# 

**Published:** 1892-06-18

**Authors:** 


No. 299. Vol. XII.] SATURDAY, JUNE 18th, 1892. [TWOPENCE.

